# Prey and habitat distribution are not enough to explain predator habitat selection: addressing intraspecific interactions, behavioural state and time

**DOI:** 10.1186/s40462-021-00250-0

**Published:** 2021-03-20

**Authors:** Alexis Grenier-Potvin, Jeanne Clermont, Gilles Gauthier, Dominique Berteaux

**Affiliations:** 1 Chaire de recherche du Canada en biodiversité nordique and Centre d’Études Nordiques, Université du Québec à Rimouski, 300 Allée des Ursulines, Rimouski, Québec G5L 3A1 Canada; 2 Département de biologie and Centre d’études nordiques, Université Laval, 2325 Rue de l’Université, Québec, Québec G1V 0A6 Canada

**Keywords:** Arctic tundra, Behavioural state, Movement, Predation risk, Resource selection, Predator-prey interactions, Spatial anchor, Territoriality, *Vulpes lagopus*

## Abstract

**Background:**

Movements and habitat selection of predators shape ecological communities by determining the spatiotemporal distribution of predation risk. Although intraspecific interactions associated to territoriality and parental care are involved in predator habitat selection, few studies have addressed their effects simultaneously with those of prey and habitat distribution. Moreover, individuals require behavioural and temporal flexibility in their movement decisions to meet various motivations in a heterogeneous environment. To untangle the relative importance of ecological determinants of predator fine-scale habitat selection, we studied simultaneously several spatial, temporal, and behavioural predictors of habitat selection in territorial arctic foxes (*Vulpes lagopus*) living within a Greater snow goose (*Anser caerulescens atlantica*) colony during the reproductive season.

**Methods:**

Using GPS locations collected at 4-min intervals and behavioural state classification (active and resting), we quantified how foxes modulate state-specific habitat selection in response to territory edges, den proximity, prey distribution, and habitats. We also assessed whether foxes varied their habitat selection in response to an important phenological transition marked by decreasing prey availability (goose egg hatching) and decreasing den dependency (emancipation of cubs).

**Results:**

Multiple factors simultaneously played a key role in driving habitat selection, and their relative strength differed with respect to the behavioural state and study period. Foxes avoided territory edges, and reproductive individuals selected den proximity before the phenological transition. Higher goose nest density was selected when foxes were active but avoided when resting, and was less selected after egg hatching. Selection for tundra habitats also varied through the summer, but effects were not consistent.

**Conclusions:**

We conclude that constraints imposed by intraspecific interactions can play, relative to prey distribution and habitat characteristics, an important role in the habitat selection of a keystone predator. Our results highlight the benefits of considering behavioural state and seasonal phenology when assessing the flexibility of predator habitat selection. Our findings indicate that considering intraspecific interactions is essential to understand predator space use, and suggest that using predator habitat selection to advance community ecology requires an explicit assessment of the social context in which movements occur.

**Supplementary Information:**

The online version contains supplementary material available at 10.1186/s40462-021-00250-0.

## Background

Habitat selection by predators is a key process shaping ecological communities through predator-prey interactions. How predators use space generates predation risk landscapes which impose costs to prey [1, 2]. At the individual scale, movements are driven by the interactions between intrinsic requirements and landscape heterogeneity, which both fluctuate at various time scales [3]. Individuals may adjust their behavioural decisions (e.g., foraging vs. other activities) to satisfy immediate needs, while they can modulate space use to adjust to environmental [4] and internal constraints [5]. Consequently, multiple spatial and temporal factors are involved in the decisions made by predators to select habitat, and thus also in the impacts of predators on prey.

The distribution of resources, land cover types, and landscape features have traditionally been used to predict predator habitat selection and to assess predation risk [6, 7]. This body of work is deeply nested in the optimal foraging [8] and ideal-free distribution theories [9], where a predator should maximize energy intake by foraging where resources are most abundant, while minimizing foraging costs. However, constraints associated to predator biology may violate a main assumption of those theories, stating that all habitat patches are equally available. For example, many predators defend a territory from conspecific intrusion, which can shape their habitat use by causing differential use of territory edges and centre [10]. Many predators are also bound to a fixed location, such as a den or nest, associated to reproduction or shelter [11]. Such spatial anchors generate space-use patterns where a declining probability of using areas farther from the focal point is expected [12]. The constraints imposed by intraspecific interactions such as territoriality and parental care likely generate conflicts with the need to maximize resource acquisition [13, 14]. Yet, surprisingly few predator habitat selection studies, at least in territorial mammals, have considered territorial competition and parental care (hereafter grouped under “intraspecific interaction constraints”) simultaneously with prey and habitat distribution.

At a fine temporal scale, habitat selection varies with behavioural motivation [15]. For example, Suraci, Frank [16] showed that lions (*Panthera leo*) avoided risky habitats when resting, but selected them when feeding. Since the behavioural state of the predator has a tremendous impact on the outcome of a predator-prey interaction [17], state-specific habitat selection is required to generate relevant predation risk landscapes. For example, the distributions of predators when they are active and resting likely contribute differently to the distributions of consumptive and non-consumptive interactions. At coarser temporal scales, temporal variation in extrinsic (e.g., resource availability) and intrinsic (e.g., life history stage) drivers can also modify space use. For example, the constraints generated by a spatial anchor associated to reproduction can be relaxed as young gain autonomy and the presence of parents at the den or nest becomes less critical [18]. Considering how habitat selection shifts across time scales is an important dimension of habitat selection research [19].

The arctic fox (*Vulpes lagopus*) is the main terrestrial predator in the arctic tundra. It feeds on many prey species, with strong top-down effect in ecosystems [20]. Habitat use of foxes is driven by factors such as prey distribution (e.g., lemmings, goose eggs [20, 21];), foraging efficiency [22], quality of denning habitats [23, 24], and territory location [25]. Habitat use and foraging strategies also vary seasonally according to fluctuating resources and cub growth [21, 26]. Fox pairs attend a den and maintain a territory throughout the year, even when reproduction fails [27]. While the biological importance of the den and territory to arctic fox is widely recognized, the influence of den and territory boundary locations on habitat selection and the associated predation risk landscape is poorly understood.

This study has two main objectives. First, we assessed the relative importance of territoriality and presence of a spatial anchor in one hand, and prey and habitat distribution in the other hand, to explain arctic fox habitat selection. Specifically, we investigated selection within summer territories in response to conspecific territory proximity, den location, goose nest density, and tundra habitats. Second, we assessed the effects of behavioural state and temporal changes in prey availability and parental investment on habitat selection. Specifically, we examined whether arctic fox habitat selection depends on whether they are active or resting, and the degree to which habitat selection varies across two phenological periods contrasted by prey availability (before and after hatching of goose eggs) and dependence on a spatial anchor (start versus end of parental care period at den).

We generated multiple predictions, as detailed in Supporting Information S1, Tables S1.1 and S1.2. In summary, for our first objective, we predicted that distance to territory edges (edges avoided by all foxes; P1a) and distance to the den (den proximity selected by reproductive foxes; P1b) explained arctic fox habitat selection, for both behavioural states and study periods. Given that active foxes likely maximize prey intake, we further predicted that prey distribution and tundra habitats also explained fox habitat selection (specifically, active foxes should select high goose nest density (P2a), avoid low quality lemming habitats (P2b), and avoid complex habitats crisscrossed by water channels impeding hunting (P2c)). For our second objective, given that the resting state is associated to energy saving or parental care, we predicted that avoidance of territory edges (all foxes) as well as selection for den proximity (reproductive foxes) would be stronger in resting than active foxes (P3a), and that high goose nest density would be avoided by resting foxes to decrease harassment (P3b). We also predicted that territory edges should remain avoided through time (P4a) but selection of den proximity should be relaxed in reproductive foxes as the summer unfolds (P4b). Finally, we predicted that selection for high goose nest density would decrease after the phenological transition (P5a), while avoidance of low-quality lemming habitats and complex habitats would remain unchanged (P5b).

## Materials and methods

### Study design

**Fig. 1:**
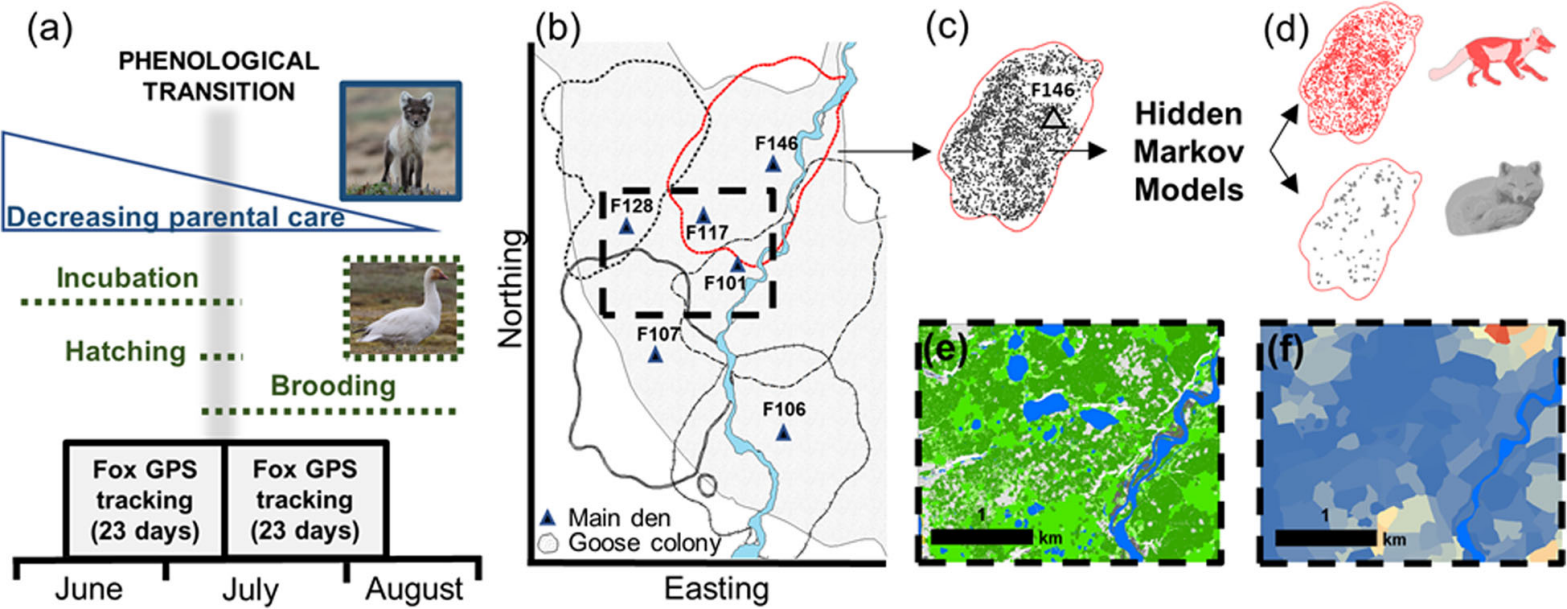
Study design used to assess arctic fox habitat selection drivers within the goose colony of Bylot Island. We used goose egg hatching and gradual emancipation of fox cubs as a phenological transition to assess temporal variations in fox habitat selection (**a**). We collected movement data during two periods from territorial foxes by tracking them with GPS collars. Territory edges were mapped by calculating a kernel utilization density for each individual while reproductive status and location of dens were assessed in the field (**b** five individual territories shown during goose incubation in 2019). We classified individual location data into active and resting behavioural states using hidden Markov models (**c** and **d**). Five habitat categories were identified in the study area from a satellite image (**e**) while goose nest density was mapped from field surveys (**f**)

We followed five steps to investigate fox habitat selection (Fig. 1). First, we took advantage of natural temporal variations to identify an important phenological transition (Fig. 1a). Specifically, we contrasted the goose incubation period when goose eggs are highly accessible to foxes, and when cubs mainly restrict their activity to the immediate den area where they are milked, to the goose brooding period when goslings disperse and are less accessible, and when cubs are gradually weaned and explore areas away from the den (Fig. 1a). The time period in which goose egg density decreases thus aligns approximately with the time period in which parental care to fox cubs strongly decreases. Second, we used solar-powered GPS collars to track foxes living in adjacent territories (Fig. 1b and c). Third, we identified with hidden Markov models the behavioural state (i.e., active or resting) associated with each GPS location (Fig. 1d). Fourth, we used high-resolution satellite data, detailed field surveys and fox GPS tracking to create a 0.5-m resolution geospatial platform which mapped tundra habitat categories (Fig. 1e), goose nest density (Fig. 1f), territory edges and main den locations. Fifth, we quantified with a resource selection function (RSF) framework [28] the relative importance of predictors of fox habitat selection for each behavioural state and period.

### Study area and ecosystem (step 1)

We worked on the southwest plain of Bylot Island (72° 53′ N, 79° 54′ W), Sirmilik National Park, Nunavut, Canada. Vegetation is characterized primarily by mesic tundra and high-center polygonal wetlands and secondarily by xeric tundra, wet meadows, gravel beds, lakes and rivers [29]. Our 52 km^2^ study area overlaps a large Greater snow goose colony and all fox locations were within 2 km of the colony boundaries (Supporting Information S2, Fig. S2.1). The colony aggregates > 20,000 goose nesting pairs [30] but goose nest density is heterogeneous due to the patchy distributions of preferred wetland habitats and snow-free patches when geese establish nesting sites in June [22]. After hatching, goose families spatially disperse up to 30 km from the colony so that goose density declines drastically in the study area [31].

Goose eggs and goslings represent a major food source for arctic foxes [23], which can consume them immediately or cache them in large quantities for later use [26]. Geese incubate for 23 days [32] and 95% of nests hatch within ±5 days of the mean hatching date [33]. Goslings are an easy prey for foxes but their vulnerability decreases as they grow up. Collared lemmings (*Dicrostonyx groenlandicus*) and especially brown lemmings (*Lemmus sibiricus*) are major prey of arctic foxes [34]. Collared lemmings occur mostly in mesic and xeric tundra and their density is relatively stable at low levels across years, whereas brown lemmings occur mostly in heterogeneous wetlands and mesic tundra and their population density peaks every 3–4 years [35]. In our study area, density of both species is low in wet meadows and gravel beds [24]. Many species of waterfowl, shorebirds, and passerines nest at low density in the study area, where they constitute incidental prey for foxes.

### Arctic fox movements (step 2)

We captured adult foxes in May–July of 2018 and 2019, following Tarroux, Berteaux [36], and equipped them with solar-powered GPS collars (RadioTag-14, Milsar, Poland). The 24-h daylight of the summer Arctic allowed us to acquire high frequency (4-min intervals) location data from May to late August.

To identify the home range of each fox during both the goose incubation and the goose brooding periods (23-day period after hatching), we assessed the 95% utilization distribution with a kernel density estimator using a subsample of 1-h interval data to minimize autocorrelation [37]. Each fox home range was bordered by the home range of at least one tracked neighbour, which allowed us to verify that individuals were territorial (e.g., Fig. 1b). All fox territories overlapped the goose colony.

### Arctic fox behavioural classification (step 3)

We used hidden Markov models (HMM) to classify fox movements in two broad behavioural states [38] (Supporting Information S3). We expected movement patterns to include an active state (long step lengths and directed travel) and a resting state (short step lengths and lots of turning), and set the model accordingly. The variable “time of the day” was included as a covariate. Separate models were built for goose incubation and goose brooding periods. The HMMs confirmed our two hypothetical behavioural states, namely active and resting, as detailed in Appendix S3, Tables S3.1 and S3.2. Although most resting steps likely represented resting behaviours, they also probably included instances where foxes were stationary but not resting.

### Georeferenced predictors (step 4)

#### Territory edges

We used the Euclidian distance to the closest territory edges (determined in step 2) as a proxy of conspecific territory proximity. Territory edges formed by the sea coast were excluded when extracting the closest territory edges since they did not relate to intraspecific competition.

#### Den locations

Fox dens were monitored yearly with camera traps, allowing identification of reproductive and non-reproductive individuals. For reproductive foxes, we considered the natal den to be the main den. For non-reproductive foxes, we used monitoring data from 2003 to 2019 to identify which den was historically most occupied by a reproductive pair, and identified this den as the main territory den. The variable “den” was measured as the Euclidian distance to the main den.

#### Habitat categories

We used a 0.5-m resolution WorldView-2 satellite image dated July 2, 2018, to generate a habitat map of the study area following the hybrid object-based approach of Chen, Pasher [39]. This supervised approach was adequate given landscape heterogeneity at different spatial scales. Following validation and ground-truthing, we obtained an overall accuracy of 98.6% for classifying water/ice/snow from land (*n*_*training*_ = 769; *n*_*validation*_ = 369), 89.4% for classifying land cover classes (*n*_*training*_ = 313; *n*_*validation*_ = 134), and 93.3% for classifying polygonal wetlands from other landscape features (*n*_*training*_ = 569; *n*_*validation*_ = 256). We merged these three classifications and used five habitat categories relevant to arctic foxes and their prey, namely mesic, wet meadows, complex wetlands, xeric, and gravel beds (Supporting Information S4, Table S4.1).

#### Goose survey

As a proxy of goose nest density, we mapped nesting goose density in each fox territory through field surveys conducted on foot during the goose incubation period (Supporting Information S5, Table S5.1). The field survey involved walking the study area with the printed satellite colour image and mapping the contours of relatively homogeneous nesting goose patches, as assessed by direct goose counts. Most geese that did not nest or failed gathered in dense groups that were not counted. Field mapping was facilitated by the abundance of landmarks (e.g., water bodies and rocks). The nesting goose count map was georeferenced in ArcGIS version 10.7 [40] to estimate patch area and to calculate nesting goose density (geese/ha). To assess the accuracy of our map, we validated it with a field survey of random plots where goose nests were systematically counted. We obtained a good correlation between nesting goose density and nest density, and thus further refer to goose nest density (Supporting Information S5). Proportions of the study area covered by goose density classes appear in Supporting Information S5, Figures S5.1 (2018) and S5.2 (2019).

#### Lemming distribution

Surveying lemmings to map their distribution across our study area was not logistically possible. Our predictions related to lemmings therefore refer to the distribution of their preferred habitats (lemming habitat preferences are summarized in step 1) rather than to their density.

### Behaviour- and period-specific habitat selection (step 5)

We quantified arctic fox habitat selection for each behavioural state (active and resting) and study period (goose incubation and brooding) using four RSF based on a use-available design at the scale of territories (third-order selection sensu Johnson [41]). We evaluated the response to distance to territory edges (meters), distance to the main den (meters), goose nest density during incubation (geese/ha), and tundra habitats (4-level categorical variable; mesic was the reference category), with individual as random factor (random intercept; Gillies, Hebblewhite [42]). GPS locations were considered as used locations (coded as 1) and five random locations were generated in the same territory for each used location to characterize availability for each individual-year [43]. RSFs were estimated from mixed logistic regression using an exponential link:

$$ {\omega}_{(x)}=\exp \left(\upbeta 1{x}_1+\upbeta 2{x}_2+\dots +\upbeta {x}_z\right) $$

where *x* is a vector of *z* covariate values, and β_1_, β_2_, ..., β_z_ are the associated regression coefficients. To satisfy the RSF assumption that the whole territory was available at every time interval [44], we retained only one GPS location every 20 min, as justified in Supporting Information S6. An interaction term between distance to the main den and reproductive status was always included as we expected the latter variable to affect den use. For all distance covariates, we considered a possible dampening effect by testing the fit of log-transformed distance relative to the fit of raw distance values, and we retained the appropriate transformation for other analyses (Supporting Information S7, Table S7.1). For each behavioural state and study period, we built a list of candidate models from the above predictors, ranging from the simplest models including only one predictor to complex models including all predictors (10, 8, 8 and 6 models were tested respectively for the fox active state during goose incubation, the resting state during incubation, the active state during brooding, and the resting state during brooding). For the active state during goose incubation, we further assessed for an interaction between the complex wetlands habitat and goose nest density, because Lecomte, Careau [22] showed that the structural complexity of wetlands decreases predation risk from foxes. We used AICc to select the most parsimonious model [45] and results from models with ∆AICc < 4 were averaged. Continuous variables were centred and standardized (mean = 0, SD = 1) to allow direct comparison among parameter estimates [46]. We tested for multicollinearity among covariates but retained all variables as *r* was always < 0.65 and the variance inflation factor < 10 [47]. We evaluated the fit of the most parsimonious models using a k-fold cross-validation with 75% of locations used as the training set and 25% as the testing set (*n* fold = 10) [48]. We report the averaged Spearman rank correlation ($\overline{r_s}$) resulting from 10 iterations. We conducted all analyses in program R version 3.6.0 [49], using lme4 to fit GLMMs [50].

## Results

Habitat selection was estimated for 8 arctic foxes (4 males, 4 females) in 2018 and 13 arctic foxes (7 males, 6 females) in 2019, which represented 14 different individuals. There were three non-reproductive foxes in both 2018 and 2019, while five foxes reproduced in 2018 and 10 in 2019. Mean (± SD) territory size was 911 (± 167) ha and 1030 (± 272) ha for the goose incubation and brooding periods, respectively. More locations were classified as active than resting during both the goose incubation (49.1 ± 9.7% vs. 40.9 ± 9.1%) and brooding (51.8 ± 9.5% vs. 40.2 ± 9.8%) periods. Only a minority of locations could not be assigned a behavioural state (Supporting Information S3, Table S3.2). After subsampling one location every 20 min, 21,619 and 15,619 classified GPS locations were available for the RSF analyses for the goose incubation and brooding periods, respectively.

### Relative importance of habitat selection predictors

**Fig. 2:**
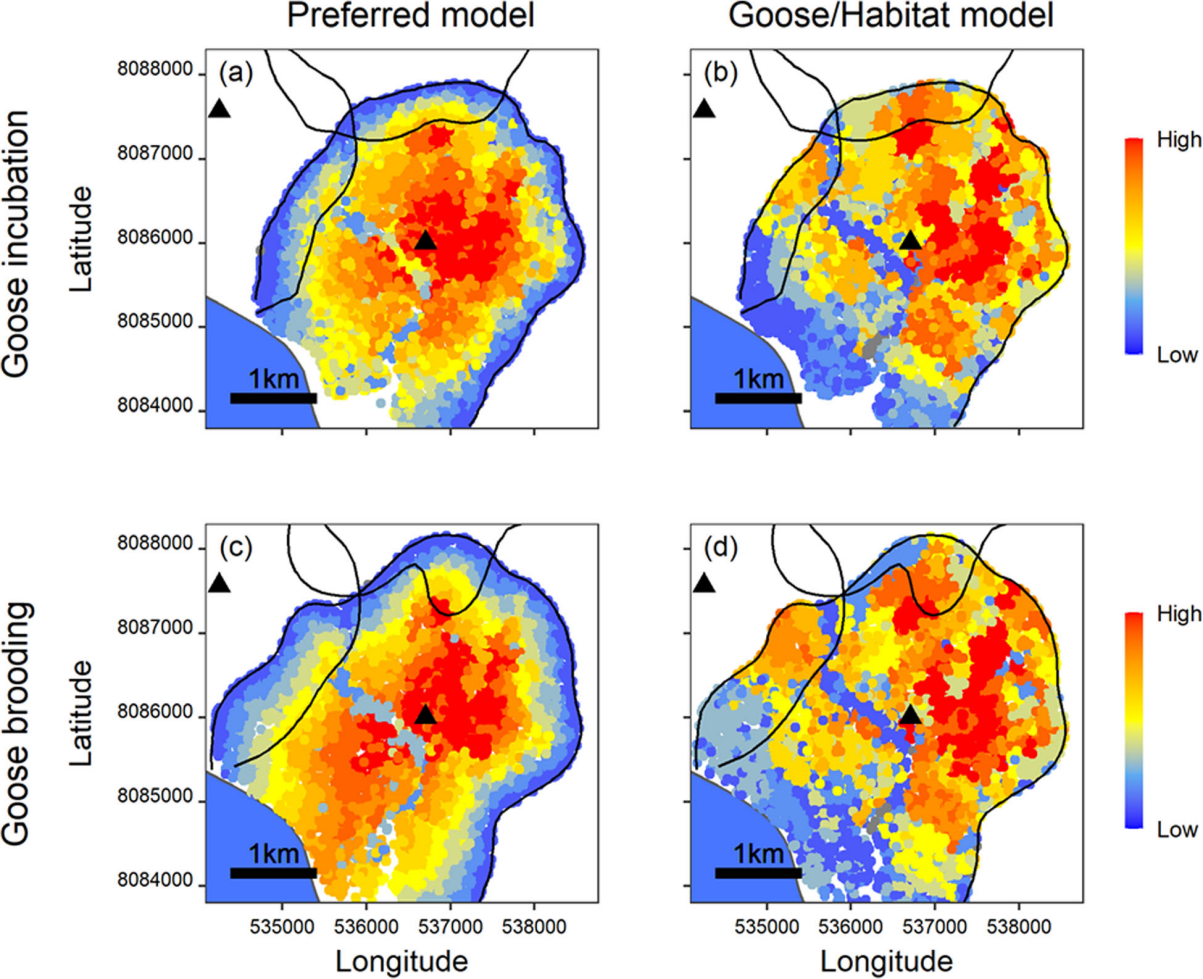
Maps showing the relative probability of selection based on third-order resource selection function (RSF), for one reproductive male arctic fox during its active state in 2019, in the snow goose colony of Bylot Island. Relative probabilities of selection are estimated with the preferred model (left column), which always includes distance to territory edges and distance to the main den, and with the model including only goose and habitat variables (right column). Territory edges of two neighbouring males are shown. The top row shows predicted relative probabilities for the goose incubation period, while the bottom row concerns the goose brooding period. Maps generated for the same fox during its resting behavioural state are presented in Supporting Information S8, Figure S8.1. Relative probabilities of selection from low to high are specific to each map, so colours should not be compared among maps. Black triangles represent main den locations

We averaged two competitive models for the RSF of active foxes during goose incubation, whereas in all other analyses a single model was preferred (Table 1). Overall, all preferred models had robust predictive power according to the k-fold cross validation (see $\overline{r_s}$ values in Table 1). Models including variables reflecting intraspecific interaction constraints (i.e., distance to territory edges and distance to the main den) always outperformed simpler models (Table 1). Still, models including only goose nest density and habitat yielded moderate to good predictive power (Table 1). Contrasting visually spatial predictions from the preferred model with those from the model including only prey and habitat revealed that omitting intraspecific interaction constraints had strong impacts on predicted predator distribution (Fig. 2 and S8.1). Among all predictors considered, territory edges had the strongest and most constant (i.e., parameter estimates similar across behavioural states and study periods) effect on habitat selection of arctic foxes (Supporting Information S8, Table S8.1). Tables S8.2, S8.3 and S8.4 show mean values of used and available locations for distance to edge, distance to den and goose nest density.

**Table 1:** Comparison between preferred models (**∆**AICc < 4) and simpler models including only habitats and goose nest density, for arctic fox resource selection functions (RSF). Models are presented for two fox behavioural states and two study periods. Note that no prediction was made regarding goose nest density for foxes resting during goose brooding (Table S1.2). Habitat selection predictors are as follows: distance to territory edges (Edge), distance to the main den (Den), goose nest density (Geese), tundra habitats (Habitat), and reproductive status (Repro)

Model	k	-LL	∆AICc	*w*_*i*_	$\overline{{\boldsymbol{r}}_{\boldsymbol{s}}}$
**(a) Fox active / Goose incubation**					
Edge + Den X Repro + Habitat + Geese X Habitat (Complex wetlands)	12	28,529.79	0.00	0.54	0.93 ± 0.02
Edge + Den X Repro + Habitat + Geese	11	28,530.95	0.32	0.46	0.93 ± 0.03
Habitat + Geese	7	29,131.90	1194.22	< 0.001	0.73 ± 0.08
**(b) Fox resting / Goose incubation**					
Edge + Den X Repro + Habitat + Geese	11	22,638.61	0.00	> 0.99	0.86 ± 0.04
Habitat + Geese	7	25,248.15	5211.07	< 0.001	0.76 ± 0.07
**(c) Fox active / Goose brooding**					
Edge + Den X Repro + Habitat + Geese	11	22,476.11	0.00	> 0.99	0.80 ± 0.07
Habitat + Geese	7	22,986.99	1013.75	< 0.001	0.57 ± 0.18
**(d) Fox resting / Goose brooding**					
Edge + Den X Repro + Habitat	10	16,630.88	0.00	> 0.99	0.97 ± 0.02
Habitat	6	18,139.79	3009.82	< 0.001	0.76 ± 0.08

*k* Number of parameters, *−LL* Negative log-likelihood, *∆AICc* Difference in AICc compared to the most parsimonious model, *w*_*i*_ AICc Weight of evidence, $\overline{r_s}$ Mean k-fold cross validation correlation coefficient ± SD

### Habitat selection across behavioural states and summer phenology

**Fig. 3:**
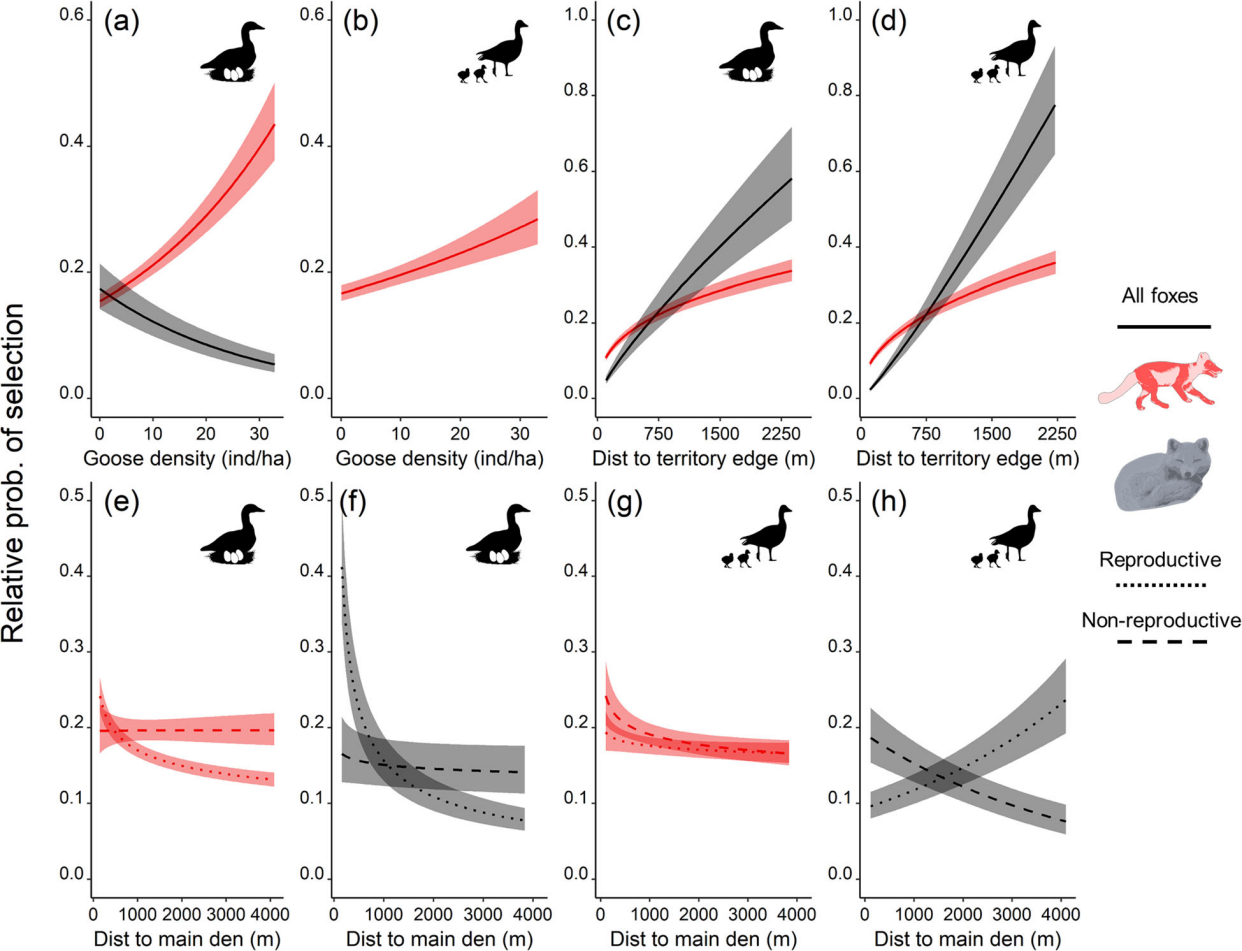
Predicted relative probability of selection (±95% CI) of arctic foxes as a function of nesting goose density (a proxy of nest density (**a** and **b**)), distance to territory edges (**c** and **d**), and distance to the main den (**e**-**h**), for two periods and two behavioural states identified by animal silhouettes. Predictions are derived from the parameter estimates of the preferred models in Table 1. The *y* and *x* axes differ between plots. No prediction was made regarding goose nest density for foxes resting during goose brooding (Table S1.2)

Habitat selection of arctic foxes clearly shifted among behavioural states and study periods (Supporting Information S8, Table S8.1). Territory edges (avoided by all foxes; P1a supported) and den proximity (selected by reproductive foxes; P1b supported) affected habitat selection of both active and resting foxes, but were less influential when foxes were active (P3a supported) (Figs. 2 and 3, and S8.1). Territory edges were similarly avoided across study periods (P4a supported; Fig. 3c and d). In reproductive foxes, selection for den proximity generally decreased as cubs gained in autonomy (i.e., during the goose brooding period), and even became negative when foxes were resting (P4b supported; Fig. 3e-h).

**Fig. 4:**
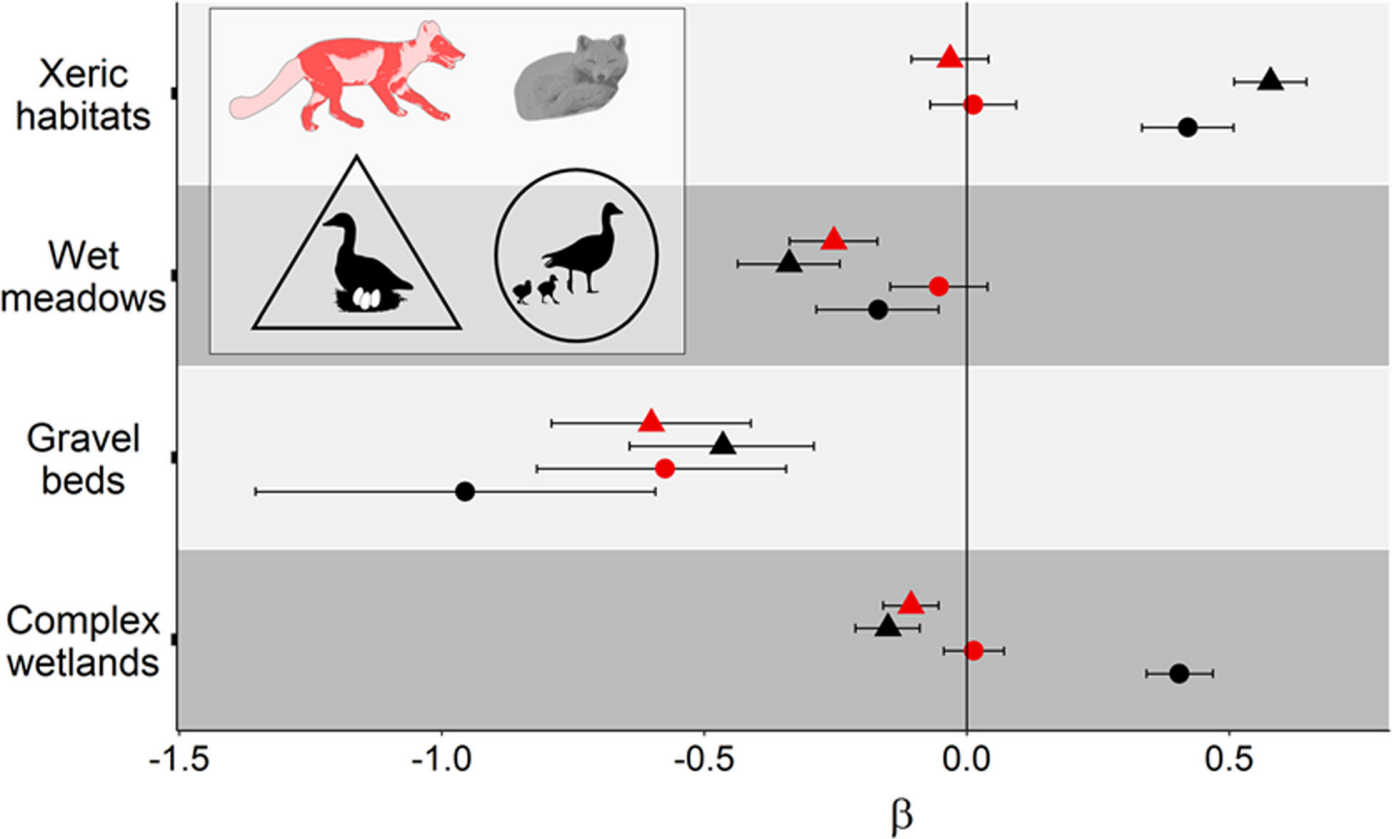
Standardized parameter estimates of tundra habitats (β ± 95% CI) from resource selection function analyses conducted on arctic foxes. Parameter estimates from the preferred models in Table 1 are presented for two periods identified by symbol shapes and two behavioural states identified by symbol colors (see animal silhouettes). Selection and avoidance are represented by positive and negative values along the *x*-axis, respectively. For clarity, parameter estimates and CI are shown on the logit scale (as estimated by logistic regression)

During the goose incubation period, foxes selected high goose nest density when active (P2a supported), but avoided it when resting (P3b supported; Fig. 3a and b). When active, the strength of selection for high goose nest density decreased from the incubation to the brooding period (P5a supported; Fig. 3a and b). Selection of various tundra habitats by foxes was more complex than predicted (Fig. 4). When active during the incubation period, foxes avoided gravel beds and wet meadows (both low-quality lemming habitats), showed no response to xeric habitats (another low-quality lemming habitat; P2b partially supported) and avoided complex wetlands (P2c supported). Across study periods, active foxes maintained a constant relation with xeric habitats (neutral selection) and gravel beds (avoidance), but shifted from avoidance to neutral selection regarding wet meadows and complex wetlands (P5b partially supported) (Fig. 4).

## Discussion

Unfolding fine-scale habitat selection is critical to reveal the complexity of predator behaviours, and ultimately how they impact their prey. Habitat selection requires individuals to make numerous decisions to cope effectively with a wide variety of internal and external factors, often involving trade-offs between individual needs and environmental heterogeneity. Our results emphasize the importance of explicitly considering intraspecific interactions, behavioural state and temporal variation in environmental features encountered by a predator. More importantly, we show that omitting constraints imposed by intraspecific interactions yields notably different and potentially misleading predator distribution compared with models that incorporate them, as illustrated by a close examination of the left and right panels in Fig. 2 and Fig. S8.1, and as supported in Table 1 by the always higher $\overline{r_s}$ values of models including intraspecific interactions. Specifically, distance to territory edges, reflecting territorial interactions with competitors, and distance to the den, reflecting a spatial anchor caused by parental care, were key factors influencing fox habitat selection. However, prey and habitat distribution also partly explained the fine-scale distribution of arctic foxes. In addition, habitat selection differed according to whether foxes were active or resting, likely reflecting tactics to maximize energy intake or minimize energy expenditures. We also found that decreasing goose egg availability and cub dependence resulted in temporal shifts in habitat selection.

### The hierarchy of predator habitat selection within the territory

Since animals should prioritise decisions affecting fitness [51], untangling the various predictors of habitat selection should inform about their hierarchy within habitat selection decision rules, and thus ultimately about the factors most important to individual fitness [8]. For instance, during the goose incubation period, avoidance of territory edges had the strongest effect on habitat selection in active reproductive foxes, while proximity to den and prey availability played similar, secondary roles. This leads to the hypothesis that negative conspecific encounters could more strongly impact survival or reproductive success than suboptimal choice of prey patches or suboptimal parental care. Interestingly, this also highlights the similar importance of parental care and prey availability, potentially because ensuring pup survival is critical for a relatively short lived species not reproducing every year. However, because the most limiting factors should affect habitat selection at large spatiotemporal scales rather than at fine scales [19], care is needed when inferring the potential fitness consequences of predictors of habitat selection from a hierarchy determined at a single spatiotemporal scale.

### The role of intraspecific interactions

It may seem counterintuitive that a territorial animal would avoid its territory borders, since the territory must be defended against conspecific intrusion. After considering potential confounding effects of prey distribution, den proximity, and territory edge proximity, we showed that avoidance of territory borders was a major determinant of habitat selection. To explain that some territorial primates avoid home range periphery, Wrangham, Crofoot [52] proposed the “Risk hypothesis” where the higher perceived risk of lethal encounters with neighbouring groups at edges should alone generate spatial avoidance. Such risk of physical aggression during a neighbour encounter also applies to canids [53, 54]. For example, Schlägel, Merrill [13] reported an avoidance of territory edges in the gray wolf (*Canis lupus*) and suggested this behaviour was associated with the increasing intraspecific mortality associated to proximity of neighbouring pack territories [53].

In arctic foxes, defending one’s territory while minimizing the costs of conspecific interactions at edges may be done in two ways, one involving olfaction and the other audition. First, scent marking at territory edges provides neighbours with long-lasting cues of territory ownership, without requiring frequent visits to edges. Second, arctic foxes of both sexes frequently bark and this can be heard over large distances, providing long-distance cues of territory ownership [55]. Also, resting foxes avoided territory edges more than did active foxes, suggesting that resting foxes minimized the potential costs of surprise encounters with neighbours. The avoidance of territory edges by a territorial predator could also be related to its foraging strategy. Indeed, arctic foxes cache large quantities of goose eggs [26], and acquiring and storing them far from territory edges could decrease pilfering by neighbours [56]. This mechanism might reinforce edges avoidance by active foxes.

The selection of den proximity by reproductive foxes confirms that parental care generates a spatial anchor during the cub raising season, when young depend on their parents for food and thermal protection. This is also supported by the fact that non-reproductive foxes respond neutrally to den location. When cubs gained energetic independence, reproductive foxes stopped selecting den proximity when active and even avoided it when resting, indicating a gradual fading of the spatial anchor at that time.

### The role of prey and habitat

The selection by active foxes of high goose nest density, and the avoidance of most low-quality lemming habitats fit the optimal foraging theory since such habitat selection likely increased prey encounters, and thus energy gained per unit of effort. Interestingly, selection of high goose nest density decreased but was still maintained after goose egg hatching. This could be explained by a habitat selection delay associated to hoarding behaviour and the recovery of unhatched eggs [21], and highlights a spatial ghost effect of the pulsed resource [57]. Given that the distribution of active goose nests affects the distribution of predation risk on other prey species [20], this ghost effect might include biased predation risk well after the goose nesting period. Such indirect and delayed community interactions between prey sharing a common predator warrant further investigations.

While the overall response to tundra habitats of active foxes likely reflected their need to collect food efficiently, temporal variations in habitat selection suggest complex relationships between predator movements and habitats. Although some poor lemming habitats (gravel beds) were always strongly avoided, other poor lemming habitats (wet meadows) or habitats where movements are difficult (complex wetlands; Lecomte, Careau [22]) were only avoided before goose egg hatching. These temporal switches suggest that decisions regarding tundra habitats were mediated by goose egg availability and (or) den dependency, although mechanisms at play are unclear. One hypothesis is that broadening their selection of habitats allowed foxes to increase the probability of encountering prey once the pulse of goose eggs had vanished and the dependence to a central place was relaxed. Theory predicts that generalist predators should use flexible strategies when facing decreasing availability of the most profitable prey [58]. This could be done by using less profitable prey [59] or by switching from a maximization of success rate tactic to a maximization of encounter rate tactic [60]. Stickney [56] demonstrated the foraging benefits of habitat selection switching when arctic foxes in Alaska faced a changing availability of prey.

### The role of behavioural state and time

The switch from avoidance of high goose nest density when resting (likely to minimize harassment risk from a large prey) to positive selection when active (likely to maximize prey intake) supports the idea that animals require different habitats in response to varying needs and motivations [44]. As reported for other predators [15, 61], considering behavioural state thus greatly helped to understand behavioural mechanisms generating fox habitat selection, and in turn also helped to gain insight on “why” animals use particular habitats [62].

Partitioning habitat selection among time periods revealed the temporal flexibility in space use and foraging strategies by an active hunting predator, likely a result of the quickly changing benefits and costs of selecting particular habitat features. Our study highlights that using temporal environmental variations (sudden changes in prey availability) or temporal within-individual variations (change in parental investment) to explain space use helps progressing towards a mechanistic understanding of predator habitat selection. Technological progress now allows intensification of tracking schedules, opening the door to increasing consideration of the many phenological transitions characterizing habitats, resources, and individual states [63].

### From habitat selection to community ecology

The existence of predator-mediated interactions between main and incidental prey is a central hypothesis explaining community structure in the arctic tundra [34]. This hypothesis involves both apparent competition, whereby one prey negatively impacts another prey through its influence on a shared predator [64], and apparent mutualism, whereby a focal prey reduces predation rate on an alternative prey because of predator saturation or selectivity [65]. Arctic foxes are often hypothesized to be the shared enemy linking the demography and spatial distribution of small tundra vertebrates [66], but mechanisms at play remain obscure or untested. Here we resolve some of these missing mechanisms.

Our demonstration that active arctic foxes select high goose nest density can explain the observations of McKinnon, Berteaux [20], who showed that predation rate of artificial bird nests increased with goose density. Avoidance of gravel beds and wet meadows by arctic foxes shows that these low-quality lemming habitats may provide refuges against fox predation for other species, which can explain why Léandri-Breton and Bêty [67] and Smith, Gilchrist [68] found that survival probability of shorebird nests was higher in those habitats. Similarly, the avoidance of complex wetlands by arctic foxes supports the suggestion of Lecomte, Careau [22] that presence of numerous water channels in this habitat hampers fox movement and accounts for the higher survival of goose nests in complex wetlands than in mesic habitats.

Constraints imposed by intraspecific interactions can also generate spatial variation in predation risk. For example, central-place foraging in African crowned eagle (*Stepahnoaetus coronatus*; Shultz and Noë [69]) and territoriality in grey wolf (Lewis and Murray [70]) affected the distribution of predation risk. Therefore, we expect the social system of arctic foxes to also generate spatial biases in predation risk in the tundra. This hypothesis could be tested experimentally in our study system by monitoring the fate of baits distributed in various parts of fox territories. As predator biology interacts with the landscape to generate patterns of predation risk [71], our understanding of top-down trophic interactions will greatly benefit from increased knowledge of the interactions between territoriality, spatial anchors, habitat and prey distribution, and behavioural state in generating spatial patterns of predation risk.

## Conclusions

This study contributes to better understanding the ecological determinants of fine-scale habitat selection and its spatial and temporal dynamics. Our results suggest that only an integrative assessment of both intraspecific interactions and prey-related predictors allows to understand why territorial predators select or avoid particular locations. We therefore recommend that further studies on habitat selection of predators, particularly those with elaborate social relations, should consider explicitly intraspecific interactions.

Our results further suggest that fox motivations regarding habitat selection strongly depend on both behavioural state and important temporal changes occurring in the ecosystem. Even though the dynamic nature of habitat selection is well recognized, it is still too rarely integrated in movement ecology studies, leading to potential noise, or even bias, when predicting animal distribution. Robust predictions of predator distribution are important when evaluating predation risk distribution or managing predators.

An integrative approach and a dynamic assessment of predator habitat selection are critical to advance our comprehension of predator-mediated processes shaping the structure and functioning of communities. For example, our results provide new insights into habitat selection by arctic foxes, a key tundra predator all over its circumpolar distribution. They provide a strong foundation to assess the effects of fox habitat selection on nesting bird distribution. More generally, our approach should provide new insights into how predator movement ecology drives spatiotemporal patterns in prey distribution.

## Supplementary Information

**Additional file 1.**

## Data Availability

GPS data are available in MoveBank study1241071371. (https://www.movebank.org/cms/webapp?gwt_fragment=page=studies,path=study1241071371).

## Abbreviations

AICc: Akaike information criterion corrected for small sample sizes
GPS: Global Positioning System
HMM: Hidden Markov Model
RSF: Resource Selection Function

## References

[1] Fortin D, Beyer HL, Boyce MS, Smith DW, Duchesne T, Mao JS (2005). Wolves influence elk movements: behavior shapes a trophic cascade in Yellowstone National Park. Ecology.

[2] Rizzari JR, Frisch AJ, Hoey AS, McCormick MI (2014). Not worth the risk: Apex predators suppress herbivory on coral reefs. Oikos.

[3] Nathan R, Getz WM, Revilla E, Holyoak M, Kadmon R, Saltz D (2008). A movement ecology paradigm for unifying organismal movement research. Proc Natl Acad Sci.

[4] Squires JR, Decesare NJ, Kolbe JA, Ruggiero LF (2010). Seasonal resource selection of Canada lynx in managed forests of the northern Rocky Mountains. J Wildl Manag.

[5] Martin J, van Moorter B, Revilla E, Blanchard P, Dray S, Quenette PY (2013). Reciprocal modulation of internal and external factors determines individual movements. J Anim Ecol.

[6] Hebblewhite M, Merrill EH, McDonald TL (2005). Spatial decomposition of predation risk using resource selection functions: an example in a wolf-elk predator-prey system. Oikos.

[7] Courbin N, Fortin D, Dussault C, Fargeot V, Courtois R (2013). Multi-trophic resource selection function enlightens the behavioural game between wolves and their prey. J Anim Ecol.

[8] Stephens DW, Krebs JR (1986). Foraging theory.

[9] Fretwell S, Lucas H (1969). On territorial behavior and other factors influencing habitat distribution in birds. Acta Biotheor.

[10] Brown JL, Orians GH (1970). Spacing patterns in mobile animals. Annu Rev Ecol Syst.

[11] Orians GH, Pearson NE, Horn DJ, Staris GR, Mitchell RD (1979). On the theory of central place foraging. Analysis of ecological systems.

[12] Schoener TW (1979). Generality of the size-distance relation in models of optimal feeding. Am Nat.

[13] Schlägel UE, Merrill EH, Lewis MA (2017). Territory surveillance and prey management: wolves keep track of space and time. Ecol Evol.

[14] Bakker ES, Reiffers RC, Olff H, Gleichman JM (2005). Experimental manipulation of predation risk and food quality: effect on grazing behaviour in a central-place foraging herbivore. Oecologia.

[15] Abrahms B, Jordan NR, Golabek KA, McNutt JW, Wilson AM, Brashares JS (2016). Lessons from integrating behaviour and resource selection: activity-specific responses of African wild dogs to roads. Anim Conserv.

[16] Suraci JP, Frank LG, Oriol-Cotterill A, Ekwanga S, Williams TM, Wilmers CC (2019). Behavior-specific habitat selection by African lions may promote their persistence in a human-dominated landscape. Ecology.

[17] Courbin N, Loveridge AJ, Fritz H, Macdonald DW, Patin R, Valeix M (2019). Zebra diel migrations reduce encounter risk with lions at night. J Anim Ecol.

[18] Studd EK, Boutin S, McAdam AG, Humphries MM (2016). Nest attendance of lactating red squirrels (*tamiasciurus hudsonicus*): influences of biological and environmental correlates. J Mammal.

[19] Rettie WJ, Messier F (2000). Hierarchical habitat selection by woodland caribou: its relationship to limiting factors. Ecography.

[20] McKinnon L, Berteaux D, Gauthier G, Bêty J (2013). Predator-mediated interactions between preferred, alternative and incidental prey in the arctic tundra. Oikos.

[21] Jepsen JU, Eide NE, Prestrud P, Jacobsen LB (2002). The importance of prey distribution in habitat use by arctic foxes (*Alopex lagopus*). Can J Zool.

[22] Lecomte N, Careau V, Gauthier G, Giroux J-F (2008). Predator behaviour and predation risk in the heterogeneous Arctic environment. J Anim Ecol.

[23] Giroux M-A, Berteaux D, Lecomte N, Gauthier G, Szor G, Bêty J (2012). Benefiting froma migratory prey: Spatio-temporal patterns in allochthonous subsidization ofan arctic predator. J Anim Ecol.

[24] Szor G, Berteaux D, Gauthier G (2007). Finding the right home: distribution of food resources and terrain characteristics influence selection of denning sites and reproductive dens in arctic foxes. Polar Biol.

[25] Angerbjörn A, Hersteinsson P, Tannerfeldt M, MacDonald DW, Silliero-Zubiri C (2004). Consequences of resource predictability in the arctic fox: two life history strategies. The biology and conservation of wild canids.

[26] Careau V, Lecomte N, Bêty J, Giroux J-F, Gauthier G, Berteaux D (2008). Hoarding of pulsed resources: temporal variations in egg-caching by arctic fox. Écoscience.

[27] Prestrud P (1992). Denning and home-range characteristics of breeding arctic foxes in Svalbard. Can J Zool.

[28] Manly BFJ, McDonald LL, Thomas DL, McDonald TL, Erickson WP (2002). Resource selection by animals: statistical design and analysis for field studies.

[29] Duclos I, Lévesque E, Gratton D, Bordelau PA (2006). Vegetation mapping of Bylot island and Sirmilik National Park: final report.

[30] Reed A, Hughes RJ, Boyd H. Patterns of distribution and abundance of greater snow geese on Bylot island, Nunavut, Canada 1983–1998. Wildfowl. 2002;53:53–65.

[31] Mainguy J, Gauthier G, Giroux JF, Duclos I (2006). Habitat use and behaviour of greater snow geese during movements from nesting to brood-rearing areas. Can J Zool.

[32] Poussart C, Larochelle J, Gauthier G (2000). The thermal regime of eggs during laying and incubation in greater snow geese. Condor.

[33] Lepage D, Gauthier G, Menu S (2000). Reproductive consequences of egg-laying decisions in snow geese. J Anim Ecol.

[34] Bety J, Gauthier G, Giroux J-F, Korpimaki E (2001). Are goose nesting success and lemming cycles linked? Interplay between nest density and predators. Oikos.

[35] Fauteux D, Gauthier G, Berteaux D (2015). Seasonal demography of a cyclic lemming population in the Canadian arctic. J Anim Ecol.

[36] Tarroux A, Berteaux D, Bêty J (2010). Northern nomads: ability for extensive movements in adult arctic foxes. Polar Biol.

[37] Fieberg J (2007). Kernel density estimators of home range: smoothing and the autocorrelation red herring. Ecology.

[38] Michelot T, Langrock R, Patterson TA (2016). Movehmm: an R package for the statistical modelling of animal movement data using hidden Markov models. Methods Ecol Evol.

[39] Chen Z, Pasher J, Duffe J, Behnamian A (2017). Mapping arctic coastal ecosystems with high resolution optical satellite imagery using a hybrid classification approach. Can J Remote Sens.

[40] ESRI (2019). Arcgis desktop 10.7. Version 10.7 ed.

[41] Johnson DH (1980). The comparison of usage and availability measurements for evaluating resource preference. Ecology.

[42] Gillies CS, Hebblewhite M, Nielsen SE, Krawchuk MA, Aldridge CL, Frair JL (2006). Application of random effects to the study of resource selection by animals. J Anim Ecol.

[43] Northrup JM, Hooten MB, Anderson CR, Wittemyer G (2013). Practical guidance on characterizing availability in resource selection functions under a use–availability design. Ecology.

[44] Beyer HL, Haydon DT, Morales JM, Frair JL, Hebblewhite M, Mitchell M (2010). The interpretation of habitat preference metrics under use-availability designs. Philos Trans Biol Sci.

[45] Burnham KP, Anderson DR (2002). Model selection and multi-model inference: a practical information-theoretic approach.

[46] Schielzeth H (2010). Simple means to improve the interpretability of regression coefficients. Methods Ecol Evol.

[47] Dormann CF, Elith J, Bacher S, Buchmann C, Carl G, Carré G (2013). Collinearity: a review of methods to deal with it and a simulation study evaluating their performance. Ecography.

[48] Boyce MS, Vernier PR, Nielsen SE, Schmiegelow FKA (2002). Evaluating resource selection functions. Ecol Model.

[49] R Development Core Team (2019). R, a language and environment for statistical computing.

[50] Bates D, Mächler M, Bolker B, Walker S. Fitting linear mixed-effects models using “lme4”. J Stat Softw. 2015;67(1):1–48.

[51] Southwood TRE (1977). Habitat, the templet for ecological strategies?. J Anim Ecol.

[52] Wrangham R, Crofoot M, Lundy R, Gilby I (2007). Use of overlap zones among group-living primates: a test of the risk hypothesis. Behaviour.

[53] Mech LD (1994). Buffer zones of territories of gray wolves as regions of intraspecific strife. J Mammal.

[54] White PCL, Harris S (1994). Encounters between red foxes (*Vulpes vulpes*): implications for territory maintenance, social cohesion and dispersal. J Anim Ecol.

[55] Frommolt K-H, Goltsman ME, Macdonald DW (2003). Barking foxes, *Alopex lagopus*: field experiments in individual recognition in a territorial mammal. Anim Behav.

[56] Stickney A (1991). Seasonal patterns of prey availability and the foraging behavior of arctic foxes (*Alopex lagopus*) in a waterfowl nesting area. Can J Zool.

[57] Yang LH, Bastow JL, Spence KO, Wright AN (2008). What can we learn from resource pulses?. Ecology.

[58] Charnov EL (1976). Optimal foraging, the marginal value theorem. Theor Popul Biol.

[59] MacArthur RH, Pianka ER (1966). On optimal use of a patchy environment. Am Nat.

[60] Rayl ND, Bastille-Rousseau G, Organ JF, Mumma MA, Mahoney SP, Soulliere CE (2018). Spatiotemporal heterogeneity in prey abundance and vulnerability shapes the foraging tactics of an omnivore. J Anim Ecol.

[61] Wilson RR, Gilbert-Norton L, Gese EM (2012). Beyond use versus availability: behaviour-explicit resource selection. Wildl Biol.

[62] Gavin TA (1991). Why ask “why”: the importance of evolutionary biology in wildlife science. J Wildl Manag.

[63] Hefty KL, Stewart KM (2019). Flexible resource use strategies of a central-place forager experiencing dynamic risk and opportunity. Mov Ecol.

[64] Holt RD (1977). Predation, apparent competition, and the structure of prey communities. Theor Popul Biol.

[65] Abrams P, Matsuda H (2005). Effects of adaptive predatory and anti-predator behaviour in a two-prey one-predator system. Evol Ecol.

[66] Lamarre JF, Legagneux P, Gauthier G, Reed ET, Bety J (2017). Predator-mediated negative effects of overabundant snow geese on arctic-nesting shorebirds. Ecosphere.

[67] Léandri-Breton D-J, Bêty J (2020). Vulnerability to predation may affect species distribution: plovers with broader arctic breeding range nest in safer habitat. Sci Rep.

[68] Smith PA, Gilchrist HG, Smith JNM (2007). Effects of nest habitat, food, and parental behavior on shorebird nest success. Condor.

[69] Shultz S, Noë R (2002). The consequences of crowned eagle central-place foraging on predation risk in monkeys. Proc Biol Sci.

[70] Lewis MA, Murray JD (1993). Modelling territoriality and wolf-deer interactions. Nature.

[71] Gaynor KM, Brown JS, Middleton AD, Power ME, Brashares JS (2019). Landscapes of fear: spatial patterns of risk perception and response. Trends Ecol Evol.

