# Supplementary material for: Prey and habitat distribution are not enough to explain predator habitat selection: addressing intraspecific interactions, behavioural state and time
Source: Mov Ecol. 2021 Mar 20;9:12. doi: 10.1186/s40462-021-00250-0 (PMC7981948; doi:10.1186/s40462-021-00250-0)
Supplement: Supplementary file 1 — Additional file 1. [file 40462_2021_250_MOESM1_ESM.pdf]

Supporting information

Prey and habitat distribution are not enough to explain predator habitat selection: addressing intraspecific interactions, behavioural state and time

Alexis Grenier-Potvin[1]*, Jeanne Clermont[1], Gilles Gauthier[2], and Dominique Berteaux[1]*

[1] Chaire de recherche du Canada en biodiversité nordique and Centre d'Études Nordiques, Université du Québec à Rimouski, 300 Allée des Ursulines, Rimouski, Québec, G5L 3A1, Canada

[2] Département de biologie and Centre d'études nordiques, Université Laval, 2325 Rue de l'Université, Québec, Québec, G1V 0A6, Canada

Appendix S1. Detailed predictions regarding the habitat selection of arctic foxes in the Bylot Island goose colony

Table S1.1. Detailed hypotheses and predictions for each habitat selection predictor used in the resource selection function analysis for arctic fox during **goose incubation** at the territory scale. The probability of selecting a location is modeled from the following spatial attributes: distance from territory edges (Edge), distance from the spatial anchor (Den), goose nest density (Geese) and physical habitat (Habitat). For the hypotheses involving Den, we always included an interaction with the reproductive status (Repro). For the hypotheses involving Prey, we tested the effect of two additive terms (Geese and Habitat) and the effect of an interaction with the complex wetland tundra habitat (Complex wetland). Pos = positive beta coefficient expected; Neg = negative beta coefficient expected; Neutral = beta coefficient expected to be close to zero; None = exploratory analysis without any *a priori* prediction; (+) or (-) = value of the beta coefficient expected to be higher (+) or smaller (-) for the resting state than for the active state; Ref = reference category for the Habitat variable; - = not applicable.

			Prediction for selection coefficient									
					Habitat							
**Hypotheses**	**Predictions**	**Habitat selection predictors**	**Distance from edge [a]**	**Distance from den[b]**	**Mesic**	**Gravel**	**Complex wetland**	**Wet meadows**	**Xeric**	**Geese**	**Interaction**	**Reference**
**(a) Fox active**												
Conspecific risk avoidance	Avoidance of territory edges	Edge	Pos	-	-	-	-	-	-	-	-	[1, 2]
Den selection	Selection for main den proximity	Den*Repro	-	Neg	-	-	-	-	-	-	Pos	[3, 4]
Prey selection	Avoidance for low quality lemming habitats and selection for areas of high goose nest density	Habitat + Geese	-	-	Ref	Neg	Neutral	Neg	Neg	Pos	-	[5-7]
Prey selection + Wetland interaction	Avoidance for low quality lemming habitats and selection for areas of high goose nest density except in complex wetland	Habitat + Geese*Complex wetland	-	-	Ref	Neg	Neutral	Neg	Neg	Pos	Neg	[8]
**(b) Fox resting**												
Conspecific risk avoidance	Avoidance of territory edges	Edge	Pos (+)	-	-	-	-	-	-	-	-	[1, 2]
Den selection	Selection for main den proximity	Den*Repro	-	Neg (-)	-	-	-	-	-	-	Pos	[3, 4]
Harassment risk avoidance	Avoidance of areas of high goose nest density	Geese	-	-	-	-	-	-	-	Neg	-	[9, 10]
Physical habitat	Selection based on physical proprieties of habitats	Habitat	-	-	Ref	None	None	None	None	-	-	[11]

[a]A positive coefficient means the probability of selecting a location increases with distance from territory edges.

[b]A negative coefficient means the probability of selecting a location decreases with distance from the den.

Table S1.2. Detailed hypotheses and predictions for each habitat selection predictor used in the resource selection function analysis for arctic fox during **goose brooding** at the territory scale. The probability of selecting a location is modeled from the following spatial attributes: distance from territory edges (Edge), distance for the spatial anchor (Den), goose nest density (Geese) and physical habitat (Habitat). For the hypotheses involving Den, we always included an interaction with the reproductive status (Repro). For the hypothesis involving Prey, we tested the effect of two additive terms (Geese and Habitat). Bold characters indicate when we expected a change in the strength of the coefficient value from the goose incubation to the goose brooding period. Pos = positive beta coefficient expected; Neg = negative beta coefficient expected; Neutral = beta coefficient expected to be close to zero; None = exploratory analysis without any *a priori* prediction; (+) or (-) = value of the beta coefficient expected to be higher (+) or smaller (-) for the resting state than for the active state; Ref = reference category for the Habitat variable; - = not applicable

			**Prediction for selection coefficient**									
					**Habitat**							
**Hypotheses**	**Predictions**	**Habitat selection predictors**	**Distance from edge** [a]	**Distance from den**[b]	**Mesic**	**Gravel**	**Complex wetland**	**Wet meadows**	**Xeric**	**Geese**	**Interaction**	**Reference**
**(a) Fox active**												
Conspecific risk avoidance	Avoidance of territory edges	Edge	Pos	-	-	-	-	-	-	-	-	[1, 2]
Den selection	Selection for main den proximity	Den*Repro	-	**Neg**	-	-	-	-	-	-	Pos	[3, 4]
Prey selection	Avoidance for low quality lemming habitats and selection of high goose nest density.	Habitat + Geese	-	-	Ref	Neg	Neutral	Neg	Neg	**Pos**	-	[5-7, 12]
**(b) Fox resting**												
Conspecific risk avoidance	Avoidance of territory edges	Edge	Pos (+)	-	-	-	-	-	-	-	-	[1, 2]
Den selection	Selection for main den proximity	Den*Repro	-	**Neg (-)**	-	-	-	-	-	-	Pos	[3, 4]
Physical habitat	Selection based on physical proprieties of habitats	Habitat	-	-	Ref	None	None	None	None	-	-	[11]

[a]A positive coefficient means the probability of selecting a location increases with distance from territory edges.

[b]A negative coefficient means the probability of selecting a location decreases with distance from the den.

Reference

1. Schlägel UE, Merrill EH, Lewis MA. Territory surveillance and prey management: Wolves keep track of space and time. Ecology and Evolution. 2017;7(20):8388-405.
2. Moorcroft PR, Lewis MA, Crabtree RL. Home range analysis using a mechanistic home range model. Ecology. 1999;80(5):1656-65.
3. Rosenberg DK, McKelvey KS. Estimation of habitat selection for central-place foraging animals. Journal of Wildlife Management. 1999;63(3):1028-38.
4. Hefty KL, Stewart KM. Flexible resource use strategies of a central-place forager experiencing dynamic risk and opportunity. Movement Ecology. 2019;7(1):1-10.
5. Jepsen JU, Eide NE, Prestrud P, Jacobsen LB. The importance of prey distribution in habitat use by arctic foxes (*Alopex lagopus*). Canadian Journal of Zoology. 2002;80(3):418-29.
6. Szor G, Berteaux D, Gauthier G. Finding the right home: Distribution of food resources and terrain characteristics influence selection of denning sites and reproductive dens in arctic foxes. Polar Biology. 2007;31(3):351-62.
7. McKinnon L, Berteaux D, Gauthier G, Bêty J. Predator-mediated interactions between preferred, alternative and incidental prey in the arctic tundra. Oikos. 2013;122(7):1042-8.
8. Lecomte N, Careau V, Gauthier G, Giroux J-F. Predator behaviour and predation risk in the heterogeneous Arctic environment. Journal of Animal Ecology. 2008;77(3):439-47.
9. Samelius G, Alisauskas RT. Foraging patterns of arctic foxes at a large arctic goose colony. Arctic. 2000;53(3):279-88.
10. Gilchrist HG, Gaston AJ, Smith JNM. Wind and prey nest sites as foraging constraints on an avian predator, the glaucous gull. Ecology. 1998;79(7):2403-14.
11. Karelus DL, McCown JW, Scheick BK, Van De Kerk M, Bolker BM, Oli MK. Incorporating movement patterns to discern habitat selection: Black bears as a case study. Wildlife Research. 2019;46(1):76-88.
12. Careau V, Lecomte N, Bêty J, Giroux J-F, Gauthier G, Berteaux D. Hoarding of pulsed resources: Temporal variations in egg-caching by arctic fox. Écoscience. 2008;15(2):268-76.

Appendix S2. Study area and contour of the snow goose colony on Bylot Island

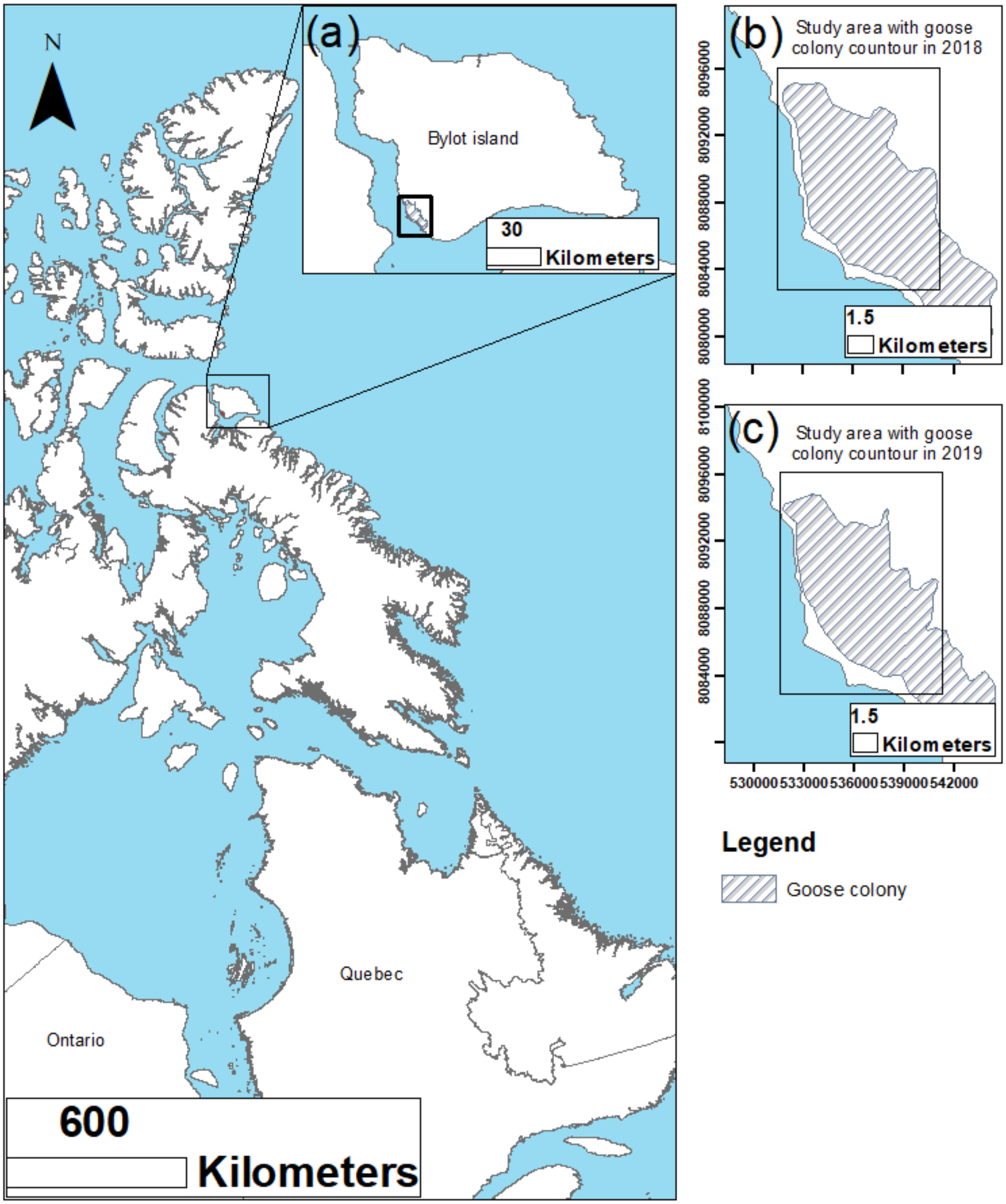

Figure S2.1. Location of the study area on Bylot Island in the Canadian High Arctic and contour of the snow goose colony in 2018 and 2019. The study area overlaps the Greater snow goose colony. Panel a) enlargement of Bylot Island; panels b) and c) enlargement of the study area showing the contours of the snow goose colony in 2018 and 2019.

Appendix S3. Hidden Markov models and results

We used hidden Markov models (HMM) with movement metrics and a time covariate, to classify fox steps in two broad behavioural states [1]. We used the step lengths (the Euclidian distance between successive locations determined 4 minutes apart) and turning angles (change in direction from the previous step) as bivariate data in HMMs. We set *a priori* parameters in the Bayesian model for two hypothetical states, namely the resting steps (no or low displacement and low directionality) and the active steps (low or high displacement and high directionality). The variable "Time of the day" was included as a covariate in the HMM because preliminary analysis of daily movement rate indicated that arctic foxes exhibited a circadian rhythm. We tested two candidate distributions for step length (gamma and Weibull) and two candidate distributions for turning angle (von Mises and wrapped Cauchy) which, added to the covariate "Time of the day", yielded 8 candidate models. To ensure numerical stability, we ran for each model 25 iterations choosing initial priors randomly within a plausible range according to the behavioural state [2]. We used the Akaike's Information Criterion (AIC) to select the most parsimonious model [3], which was then used with the Viterbi algorithm to classify the most likely state to each step [4]. The behavioural classification was done independently for the goose incubation and brooding periods. As we used GPS locations rather than steps to assess arctic fox habitat selection (see *2.6 Behaviour- and period-specific habitat selection (step 5)*), we only retained for RSFs those locations for which the previous and next steps were assigned the same state. We used the moveHMM package to prepare the data, to fit the HMMs, and to classify GPS locations [1].

Reference

1. Michelot T, Langrock R, Patterson TA. Movehmm: An R package for the statistical modelling of animal movement data using hidden Markov models. Methods in Ecology and Evolution. 2016;7(11):1308-15.
2. Karelus DL, McCown JW, Scheick BK, Van De Kerk M, Bolker BM, Oli MK. Incorporating movement patterns to discern habitat selection: Black bears as a case study. Wildlife Research. 2019;46(1):76-88.
3. Burnham KP, Anderson DR. Model selection and multi-model inference : A practical information-theoretic approach. 2nd ed. ed. New York: Springer; 2002 2002. xxvi, 488 p. p.
4. Langrock R, King R, Matthiopoulos J, Thomas L, Fortin D, Morales JM. Flexible and practical modeling of animal telemetry data: hidden Markov models and extensions. Ecology. 2012;93(11):2336-42.

Table S3.1. Step length and angle distribution parameters of the top two-state hidden Markov model for arctic fox during the goose incubation and the goose brooding periods. The step lengths are modeled with a Weibull distribution, and the turning angles with a wrapped Cauchy distribution based on a 4-minute interval GPS location dataset. As angles are circular quantities, circular mean is necessary to express a measure of central tendency. A circular mean approaching 0 indicates low turning angles (animals are generally moving forward), while a circular mean approaching 3.14 indicates high turning angles (animals are generally turning backward). Angle concentration is a measure of dispersion, where 0 means uniform distribution and 1 means complete concentration in one direction.

Behavioural state	Goose incubation period		Goose brooding period	
	Resting<br>*n* = 50,747	Active<br>*n* =63,794	Resting<br>*n* =38,536	Active<br>*n* =53,666
Step mean (meters)	9.9	230.0	9.6	225.9
Step SD (meters)	10.7	132.8	10.5	132.8
Angle circular mean (radians)	3.14	0.03	3.13	0.02
Angle concentration	0.36	0.43	0.35	0.46

Table S3.2. Proportion of arctic fox locations by behavioural state, based on 4-minute interval GPS location data during the goose incubation and the goose brooding periods (mean ± SD). The active behavioural state is characterized by low to high displacement and high directionality. The resting behavioural state is characterized by no or low displacement and low directionality. "Transition" represents locations for which the previous and next behavioural states differ. "Timeout" identifies instances when the GPS tried to calculate a geographic location but no satellites were available to do so. In the relatively flat tundra, these instances mostly reflect times when foxes were inside their den, as preliminary analysis showed that most previous and next locations of a subset of "timeout" locations were in the close vicinity of a den.

	Goose incubation period<br>*n* = 21 fox-summers				Goose brooding period<br>*n* = 20 fox-summers			
Behavioural state	Active	Resting	Transition	Timeout	Active	Resting	Transition	Timeout
Proportion of locations	49.1% (± 9.7)	40.9% (± 9.1)	4.6% (± 0.8)	5.4% (± 7.7)	51.8% (± 9.5)	40.2% (± 9.8)	4.6% (± 0.8)	3.4% (± 5.5)

Appendix S4. Habitat classification

Table S4.1. Physical habitat classes used to study arctic fox habitat selection. The study area was classified based on vegetation composition, soil moisture, and density of water channels. We used previous work from Bylot Island [1-3] to set *a priori* classes biologically relevant for arctic foxes, and give in the right column the corresponding vegetation compositions and morphometric descriptions used by this previous research.

Class	Definition	Correspondence with previous work
Mesic	Typically <50% barren ground. Moist soil with a diverse vegetation composition (moss, herbaceous, *Salix* sp., *Cassiope tetragona*, lichens, etc). Complex microtopography characterized by presence of hummocks and mudboils. Hummocks are concave and mostly vegetated structures 15-50 cm wide and 10-30 cm high. Mudboils are 30-50 cm wide cryoactive round surfaces of bare ground. Dry to muddy soil.	Correspondence with vegetation composition of Duclos [2] and Duclos, Lévesque [3]: Graminoid moist meadow, Moist meadow, Moist shrub tundra, Grass mesic meadow, Shrub heath tundra (*Dryas integrifolia*), Shrub heath tundra (*Cassiope tetragona*).
Wet meadows	<10% barren ground. Vegetation dominated by mosses and, to a lesser extent, herbaceous plants. Highly productive. Microtopography is flat. Soil is waterlogged and vegetation commonly flooded when raining. Not characterized by water channels. Include low-center polygon with collapsed or degraded rims.	Correspondence with vegetation composition of Duclos [2] and Duclos, Lévesque [3]: Graminoid wet meadows.<br><br>Correspondence with morphometric description of Massé, Rochefort [1]: Wet polygon (low complexity), Lake polygon (low complexity).
Xeric	Typically >75% barren ground. Dry ground covered mainly by small rocks (granulometry 1-5 cm) and secondarily by mud or exposed soil. Sparse vegetation dominated by *Dryas* sp., *Salix* sp., lichens and *Cassiope tetragona*.	Correspondence with vegetation composition of Duclos [2] and Duclos, Lévesque [3]: Shrub heath tundra (*Dryas integrifolia*), Shrub forb tundra, Forb-Salix barrens.
Gravel beds	Mostly riverbanks. Characterized by almost 100% barren ground with rock of variable sizes (granulometry 1-15 cm) in a matrix of fine sediment. Little or no vegetation.	
Complex wetlands	Mostly high-center polygons. May include low-center polygons when rims are large and clearly defined. Complex mosaic of water bodies, mesic habitats, and moss carpet.	Correspondence with morphometric description of Massé, Rochefort [1]: Polygon channels, Wet polygon (high complexity), Lake polygon (high complexity).

Reference

1. Massé H, Rochefort L, Gauthier G. Carrying capacity of wetland habitats used by breeding greater snow geese. The Journal of Wildlife Management. 2001;65(2):271-81.
2. Duclos I. Milieux mésiques et secs de l'île Bylot, Nunavut (Canada) : Caractérisation et utilisation par la grande oie des neiges [Thesis]: Université du Québec à Trois-Rivières; 2002.
3. Duclos I, Lévesque E, Gratton D, Bordelau PA. Vegetation mapping of Bylot island and Sirmilik National Park: Final report. Parks Canada, Iqaluit, Nunavut. Unpublished report. 2006:101pp.

Appendix S5. Goose survey and validation

Goose density validation

Apart from lemmings, the main food resource for foxes during the goose incubation period is goose eggs, as foxes very rarely prey on adult geese. We validated our goose density map using systematic nest counts performed during the annual monitoring of the Bylot Island goose colony. Goose nests are searched and counted in a variable number of random plots located in mesic and wetland habitats. Plots are larger in mesic (4-ha, 200m x 200m) than in wetland habitat (1-ha, 100m x 100m) because of the lower density of goose nests in the former. Specifically, we walked along several parallel transects across the plots to find the nests. Goose nests are easy to find on the tundra (even when females are off the nest) due to their large size and the presence of down at the nest. We validated our goose density map using 13 plots each year (2018: $n_{\text{mesic}} = 6$, $n_{\text{wetland}} = 7$; 2019: $n_{\text{mesic}} = 5$ and $n_{\text{wetland}} = 8$). We extracted within each monitored plot the mean density values generated from our map to assess the correspondence with the systematic plot counts. We found a good correlation between the two ($r_{\text{Spearman}} = 0.62$, $p < 0.01$, n = 26), thus confirming that our nesting goose density map represented well the availability of goose nests for foxes.

Table S5.1. Starting and ending dates for the goose incubation and brooding periods. End of incubation corresponds to the estimated median goose hatching date (n = number of nests) obtained from the annual Bylot Island goose monitoring (G. Gauthier, unpublished data) and incubation start is 23 days (= incubation length) earlier. The brooding period starts the day after the end of the incubation period and lasts for 23 days according to our study design (brooding duration is actually longer, but fox habitat selection was only studied during the first 23 days to obtain a temporally balanced design). Field surveys for mapping nesting goose density were done from June 30 to July 4 in 2018, and from July 1-6 in 2019. Note that fox cubs require high parental investment during goose incubation, but much lower parental investment during goose brooding when they gradually become thermally independent and learn to forage by themselves.

Year	Start of goose incubation period	End of goose incubation period	Start of goose brooding period	End of goose brooding period
2018	June 19	July 11(n =106)	July 12	August 3
2019	June 12	July 4 (n =180)	July 5	July 27

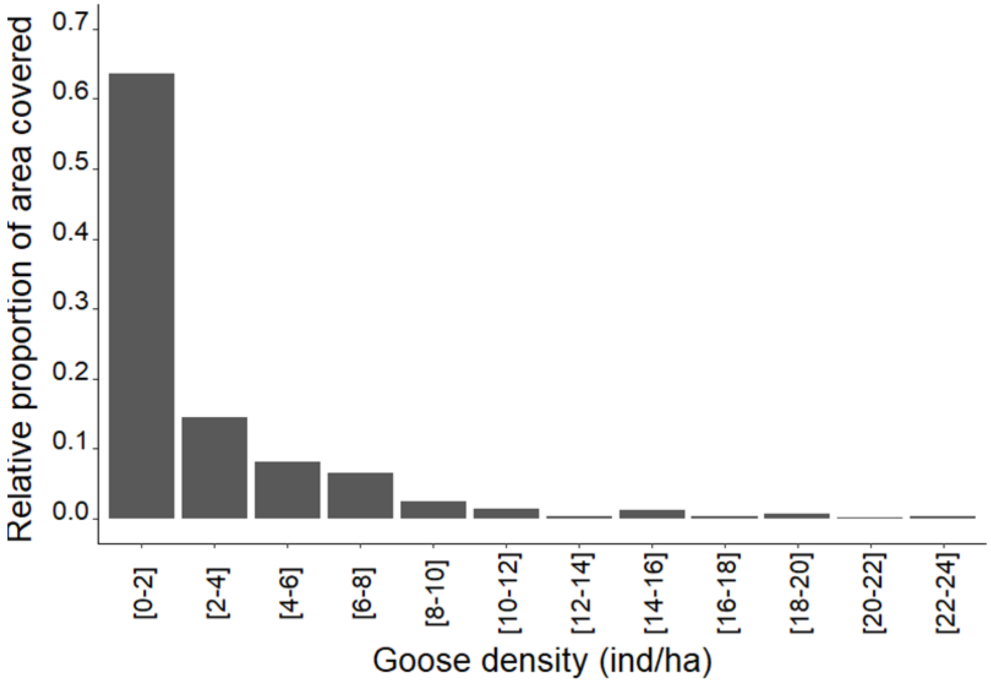

Figure S5.1. Relative proportion of the study area covered by each goose density class for the 6,438 ha area surveyed in 2018 to generate the goose density map. Note that the [0-2] class includes areas surveyed in the study area but located outside the goose colony. Also note that in 2018 the median patch area [interquartile range] was 11.0 ha [7.2; 17.0]).

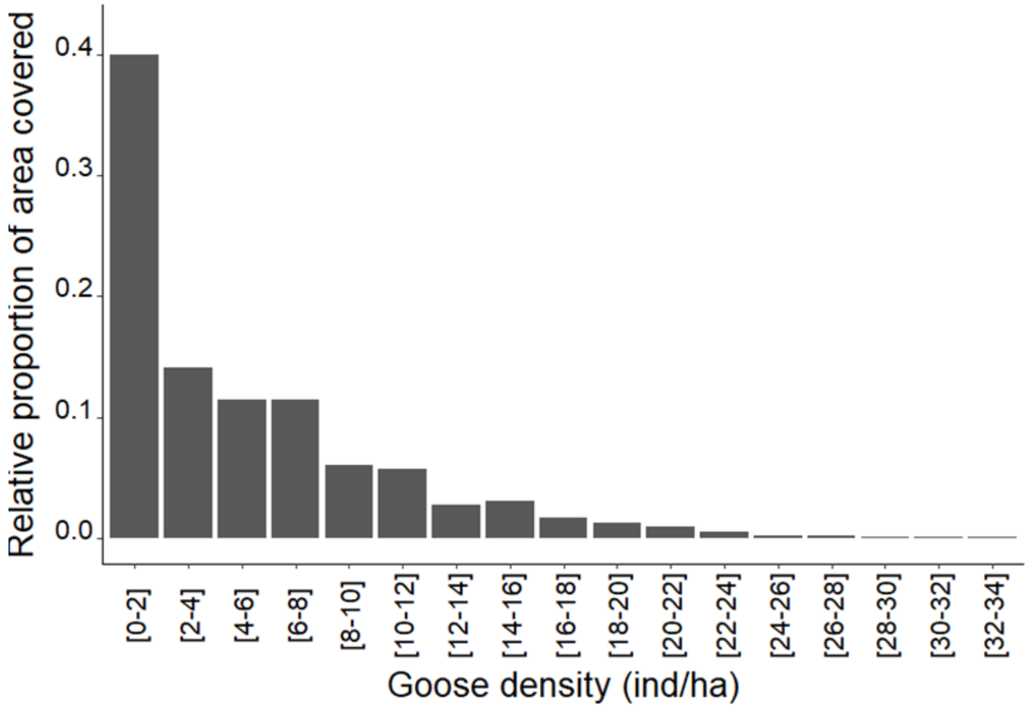

Figure S5.2. Relative proportion of the study area covered by each goose density class for the 5,692 ha area surveyed in 2019 to generate the goose density map. Note that the [0-2] class includes areas surveyed in the

study area but located outside the goose colony. Also note that in 2019 the median patch area [interquartile range] was 7.6 ha [5.0; 11.8]).

Appendix S6. Justification of 20-minute subsampling

An important assumption of RSFs is that most locations in the territory are available at each step. To meet this assumption, we subsampled our 4-minute interval dataset to allow individuals to travel the equivalent of an average territory radius between locations. A mean arctic fox territory radius was 1500 m (n = 21 fox-summers). After dividing this distance by the third quartile value (306 m) of 4-min step lengths calculated from locations of active foxes, we found that a fox can cover a territory radius in 4.90 x 4 min, which we rounded to 20 min. We used the third quartile value rather than the mean, median or highest step length in order to reflect the traveling capacity of foxes while avoiding the assumption that foxes can regularly move as fast as suggested by the highest measured speed values. Note that even if step lengths are considerably shorter in resting than in active foxes, we assumed that a resting individual also had the possibility to cover a territory radius within 20 min, if deciding to do so.

Appendix S7. Model selection evaluating the effect of log-transforming distance variables

Table S7.1. List of candidate arctic fox RSF models assessing log-transformation of the variables Distance from territory edges (Edge) and Distance from the spatial anchor (Den). The effect of the log-transformation is shown for Edge and Den through the negative maximum log-likelihood (-LL), the difference in Akaike information criterion corrected for small sample sizes with the most parsimonious model (ΔAIC_c) and the AIC_c weight of evidence (w_i). All models had k=11 parameters and included Edge (raw or log-transformed), Goose nest density (not shown), Physical habitat (not shown), and Den (raw or log-transformed) with its interaction with reproductive status of individuals. Covariates are further defined in the main text (section *2.5 Georeferenced predictors (step 4)*). Model selection was done within each goose breeding period (incubation and brooding) and behavioural state (active and resting). The lines in bold under "Transformations" identify top-ranked models and these transformations (or lack of) were applied in the state- and period-specific resource selection analyses described in section *2.6 Behaviour- and period-specific habitat selection (step 5)*.

Period	Behavioural state	Distance variable	Transformation	-LL	ΔAICc	w_i
Goose incubation	Active	Edge	none [raw values]	28862.17	565.43	<0.001
			**log [1+ raw values]**	28579.46	0.00	>0.999
Goose incubation	Active	Den	none [raw values]	28862.17	138.76	<0.001
			**log [1+ raw values]**	28792.79	0.00	>0.999
Goose incubation	Resting	Edge	none [raw values]	24124.15	1575.39	<0.001
			**log [1+ raw values]**	23336.45	0.00	>0.999
Goose incubation	Resting	Den	none [raw values]	24124.15	1590.01	<0.001
			**log [1+ raw values]**	23329.14	0.00	>0.999
Goose brooding	Active	Edge	none [raw values]	22740.31	529.54	<0.001
			**log [1+ raw values]**	22475.54	0.00	>0.999
Goose brooding	Active	Den	none [raw values]	22740.31	1.90	0.28
			**log [1+ raw values]**	22739.36	0.00	0.72
Goose brooding	Resting	Edge	none [raw values]	17229.90	1201.32	<0.001
			**log [1+ raw values]**	16629.24	0.00	>0.999
Goose brooding	Resting	Den	**none [raw values]**	17229.90	0.00	>0.999
			log [1+ raw values]	17279.65	99.51	<0.001

Appendix S8. Resource selection function analysis results

Table S8.1. Parameter estimates of mixed-effect best (top-ranked or averaged) models of resource selection function (RSF) for arctic foxes in their active and resting behavioural states during the goose incubation (n=21 fox-summers) and brooding (n=20 fox-summers) periods (see Table 1). Only the terms included in the best model appear in the corresponding analysis table. Covariates are defined in the main text (section *2.5 Georeferenced predictors (step 4)*). Standardized selection coefficients (β) and their 95% confidence intervals (95% CI) are shown. "Mesic habitat" and "Reproductive" are the reference categories for tundra habitat and Reproductive (Rep.) status, respectively. Coefficients in bold indicate where 95% confidence intervals exclude zero.

	Goose incubation period				Goose brooding period			
Behavioural state	Active		Resting		Active		Resting	
Variable	β	95% CI	β	95% CI	β	95% CI	β	95% CI
(Intercept)	**-1.43**	[-1.36;-1.50]	**-1.83**	[-1.62;-2.05]	**-1.56**	[-1.49;-1.64]	**-1.97**	[-1.79;-2.15]
Wet meadows	**-0.25**	[-0.17;-0.34]	**-0.34**	[-0.24;-0.44]	-0.05	[0.04;-0.15]	**-0.17**	[-0.05;-0.29]
Complex wetlands	**-0.11**	[-0.05;-0.16]	**-0.15**	[-0.09;-0.21]	0.01	[0.07;-0.04]	**0.41**	[0.47;0.34]
Xeric habitats	-0.03	[0.04;-0.11]	**0.58**	[0.65;0.51]	0.01	[0.09;-0.07]	**0.42**	[0.51;0.33]
Gravel beds	**-0.60**	[-0.41;-0.79]	**-0.47**	[-0.29;-0.64]	**-0.58**	[-0.34;-0.82]	**-0.96**	[-0.59;-1.36]
Goose density x Complex wetlands	0.03	[0.07;-0.01]	-	-	-	-	-	-
Goose density	**0.17**	[0.19; 0.14]	**-0.19**	[-0.16;-0.22]	**0.09**	[0.11; 0.06]	-	-
Log [Edge]	**0.38**	[0.41; 0.35]	**0.85**	[0.89; 0.81]	**0.46**	[0.49; 0.42]	**1.18**	[1.23; 1.13]
Log [Den]	0.00	[0.06;-0.05]	-0.04	[0.02;-0.10]	**-0.07**	[-0.03;-0.11]	-	-
Log [Den] X Rep. status	**-0.14**	[-0.08;-0.20]	**-0.36**	[-0.30;-0.43]	0.04	[0.09;-0.01]	-	-
Den	-	-	-	-	-	-	**-0.18**	[-0.12;-0.24]
Den X Rep. status	-	-	-	-	-	-	**0.36**	[0.43; 0.29]
Rep. status	**-0.17**	[-0.10;-0.25]	-0.04	[0.1;-0.17]	-0.06	[0.01;-0.13]	-0.01	[0.11;-0.12]

Table S8.2. Mean values with 95% CI of **distance to the edge** (meters) for locations used by and available to arctic foxes. Data are split by period and behavioural state. Values for used locations were calculated from all fox locations used in the RSF, and values for available locations were calculated from all random locations used in the RSF.

Period	Behavioural state	Used/available	Mean	95% CI
Goose incubation	Active	Used	683.3	[674.6; 692.0]
		Available	592.4	[588.0; 596.8]
Goose incubation	Resting	Used	800.5	[791.3; 809.7]
		Available	582.7	[578.0; 587.7]
Goose brooding	Active	Used	743.6	[733.8; 753.4]
		Available	636.8	[631.7; 642.0]
Goose brooding	Resting	Used	872.7	[862.3; 883.1]
		Available	635.7	[629.9; 641.5]

Table S8.3. Mean values with 95% CI of **distance to the den** (meters) for locations used by and available to arctic foxes. Data are split by period, behavioural state and reproductive status. Values for used locations were calculated from all fox locations used in the RSF, and values for available locations were calculated from all random locations used in the RSF. For reproductive status, R and NR indicate reproductive and non-reproductive.

Period	Behavioural state	Reproductive status	Used/available	Mean	95% CI
Goose incubation	Active	R	Used	1339.8	[1321.9; 1357.7]
			Available	1453.9	[1445.9; 1461.9]
Goose incubation	Resting	R	Used	1215.6	[1191.0; 1240.2]
			Available	1451.6	[1442.9; 1460.2]
Goose incubation	Active	NR	Used	1465.7	[1436.5; 1495.0]
			Available	1498.2	[1484.3; 1512.1]
Goose incubation	Resting	NR	Used	1269.1	[1245.3; 1292.9]
			Available	1441.4	[1428.0; 1454.8]
Goose brooding	Active	R	Used	1465.4	[1443.6; 1487.2]
			Available	1582.6	[1571.8; 1593.3]
Goose brooding	Resting	R	Used	1470.5	[1446.1; 1494.8]
			Available	1578.4	[1566.0; 1590.7]
Goose brooding	Active	NR	Used	1508.5	[1479.9; 1537.2]
			Available	1629.0	[1614.7; 1643.3]
Goose brooding	Resting	NR	Used	1386.0	[1361.1; 1411.0]
			Available	1614.5	[1599.7; 1629.3]

Table S8.4. Mean values with 95% CI of **goose nest density** (geese/ha) for locations used by and available to arctic foxes. Data are split by period and behavioural state. Values for used locations were calculated from all fox locations used in the RSF, and values for available locations were calculated from all random locations used in the RSF. Note that no prediction was made regarding goose nest density for foxes resting during goose brooding (Table S1.2).

Period	Behavioural state	Used/available	Mean	95% CI
Goose incubation	Active	Used	5.69	[5.56; 5.82]
		Available	4.70	[4.65; 4.76]
Goose incubation	Resting	Used	3.72	[3.59; 3.84]
		Available	4.87	[4.82; 4.93]
Goose brooding	Active	Used	4.96	[4.82; 5.10]
		Available	4.46	[4.40; 4.52]

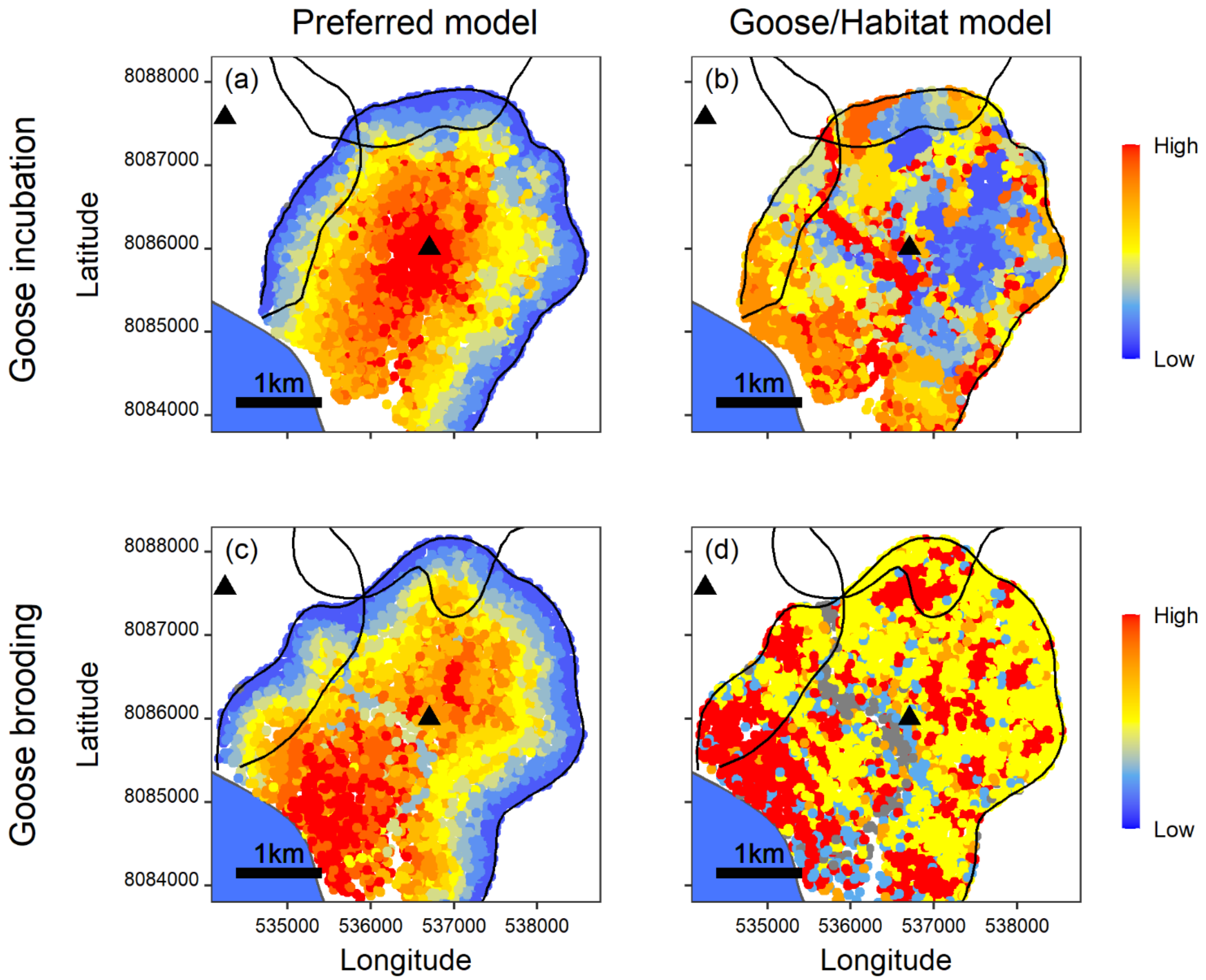

Figure S8.1. Maps showing the relative probability of selection based on third-order resource selection function (RSF), for one reproductive male arctic fox during its resting state in 2019, in the snow goose colony of Bylot Island. Relative probabilities of selection are estimated with the preferred model (left column), which always includes distance to territory edges and distance to the main den, and with the model including only goose and habitat variables (right column). Territory edges of two other males are presented. The top row shows predicted relative probabilities for the goose incubation period, while the bottom row concerns the goose brooding period. Relative probabilities of selection from low to high are specific to each map, so colours should not be compared among maps. Black triangles represent main den locations.
